# A Study of an Online Tracking System for Spark Images of Abrasive Belt-Polishing Workpieces

**DOI:** 10.3390/s23042025

**Published:** 2023-02-10

**Authors:** Jian Huang, Guangpeng Zhang

**Affiliations:** 1 School of Mechanical and Precision Instrument Engineering, Xi’an University of Technology, Xi’an 710048, China; 2 School of Computer Science, Xijing University, Xi’an 710123, China

**Keywords:** spark images, YOLO5, target detection, abrasive belt grinding

## Abstract

During the manual grinding of blades, the workers can estimate the material removal rate based on their experiences from observing the characteristics of the grinding sparks, leading to low grinding accuracy and low efficiency and affecting the processing quality of the blades. As an alternative to the recognition of spark images by the human eye, we used the deep learning algorithm YOLO5 to perform target detection on spark images and obtain spark image regions. First the spark images generated during one turbine blade-grinding process were collected, and some of the images were selected as training samples, with the remaining images used as test samples, which were labelled with LabelImg. Afterwards, the selected images were trained with YOLO5 to obtain an optimisation model. In the end, the trained optimisation model was used to predict the images of the test set. The proposed method was able to detect spark image regions quickly and accurately, with an average accuracy of 0.995. YOLO4 was also used to train and predict spark images, and the two methods were compared. Our findings show that YOLO5 is faster and more accurate than the YOLO4 target detection algorithm and can replace manual observation, laying a specific foundation for the automatic segmentation of spark images and the study of the relationship between the material removal rate and spark images at a later stage, which has some practical value.

## 1. Introduction

Turbine compressors have been widely used in petrochemical, metallurgical, and aviation fields. The blade is a crucial part of turbine power systems with complex structures, and its machining quality directly affects the operational performance and operating efficiency of the turbine power system. Many turbine blades are currently machined by manual grinding. When grinding, workers observe the characteristics of the grinding sparks and rely on experience to judge the material removal rate and control the processing of the blades. Experienced workers can machine a workpiece very quickly. However, this method is challenging to pass on, and master workers with extensive blade machining experience tend to be older. Additionally, due to poor working conditions, most young people are reluctant to take up this work.

Many scholars have studied these problems in order to solve them and achieve automatic control of the blade machining process. Qi, J.D. et al. [1] proposed a method for monitoring the material removal rate of abrasive belt grinding based on an improved convolutional neural network (CNN). A multisensor fusion grinding system for sound and vibration was established. Pandiyan, V. et al. [2] introduced a tool condition monitoring and prediction system and developed an abrasive belt wear prediction model based on a genetic algorithm (GA) and support vector machine (SVM). Pandiyan V et al. [3] reported that AI algorithms are not fully applied in abrasive processing and prediction for online monitoring and modelling of research trends. Gao, K. et al. [4] proposed an acoustic sensing and machine-learning-based material removal rate model. The k-fold XGBoost algorithm was used to train the collected acoustic signals, and the algorithm reduced the mean absolute percentage error (MAPE) of material removal to 4.373% compared to support vector regression. A new trained and optimised k-fold extreme gradient enhancement (k-fold-boost) algorithm was integrated into the material removal (Mr) model. Experimental results show that the model predictions agree with the measured values. The mean absolute percentage error (MAPE) of the material removal rate evaluated by the model was 4.373%, outperforming other models by 6.4 to 8.72%.

Today, with the continuous development of computer technology and the improvement of hardware performance, machine learning methods have been developed rapidly, and many research hotspots have emerged, including target detection algorithms. In the present study, we attempted to use this method for real-time tracking and detection of spark images. The most commonly used target detection algorithms are the YOLO algorithms [5,6,7], including the YOLOv4 algorithm and optimal speed and accuracy of object detection; [8] the SSD algorithm [9]; and RCNN [10,11,12]. Kaiming He presented MASK R-CNN, a conceptually simple, flexible, and general framework for object instance segmentation [13]. Depending on the process, algorithms can be divided into two categories: one-stage algorithms and two-stage algorithms. YOLO and the SSD algorithm belong to the one-stage algorithm category and based on anchor classification and adjusts the bounding box. Fast-RCNN and Faster-RCNN belong to the two-stage algorithm category and detect targets in two steps using a dedicated module that generates candidate frames, finds the foreground, and adjusts the bounding box to detect the target. Each type of algorithm has its pros and cons, with two-stage detection being slow and accurate and one-stage detection being fast and inaccurate. However, the YOLO algorithm has evolved from YOLO1 to YOLO5, with speed and accuracy gradually improving, and YOLO5 has become the most advanced target detection algorithm currently available. The core philosophy behind the YOLO algorithms is to transform target detection into a regression problem solver based on a separate end-to-end network from the input of the original image to the output of the object position and category.

Many scholars have studied and improved aim detection algorithms, for example, Fu, L. et al. [14] An adaptive spatial pixel-level feature fusion network, the ASPFF network, was proposed to detect pedestrian targets on a multiscale feature layer by fusing complementary information from visible and thermal infrared images. Lian, J. et al. [15] proposed a method for detecting small targets in traffic scenes based on attentional feature fusion. Wenkel, S. et al. [16] proposed a method for finding the best performance point of the model based on a fairer selection of confidence score thresholds. Wang, J. et al. [17] proposed a method for target localization and behaviour recognition based on visual images. The YOLOv3 model was used to detect the acquired images, accomplishing improved detection of experimental targets. Arunabha, M. et al. [18] used an improved YOLOv4 algorithm to solve the problems of dense distribution, irregular morphology, multiscale target classes, and texture similarity in plant disease detection.

We have conducted research on the abrasive belt blasting workpiece process with our team for many years. Ren, L.J. et al. [19] studied spark images generated during the abrasive belt blasting process, extracted characteristics including spark image area and illumination, and studied the relationship between spark field characteristics and the material removal rate. Wang Nina et al. [20,21] studied the characteristics of sound and spark images during the abrasive belt grinding process, fused the signals of images and sound, and established a material removal rate model.

The following problems were encountered: (1) The spark information obtained due to the change of the environment in the actual grinding process may contain errors. (2) Under dark conditions, the spark image was difficult to distinguish. (3) The spark image involved organic machine tools, workpieces, and other parts that were difficult to distinguish and identify. (4) The spark image was processed after complete processing was completed, and a real-time detection system for the spark image could not be established.

In this study, the goal was to build a real-time spark image tracking and detection system to accurately detect the spark image area in a complex environment and quickly identify spark images. The ultimate goal was to establish a spark image and material removal rate prediction model that can realize automatic processing control of the workpiece, which is an area requiring further research.

While Ren LJ and Wang Nina have studied spark images during abrasive belt grinding, online tracking and real-time processing of the abrasive belt grinding process have not been achieved. Accordingly, the aim of this study was the online tracking and detection of spark images using the current state-of-the-art YOLO5 algorithm to quickly and accurately identify and detect spark images. The remainder of this paper is organised as follows. Section 2 describes the experimental setup process and briefly introduces the proposed spark image acquisition method for the abrasive belt grinding process. Section 3 introduces the preprocessing and labelling of the images, and Section 4 describes the main methods used. Section 5 presents the experiments, the training and testing results of the model, and a comparison of the results obtained by different algorithms. In Section 6, we present our conclusions and possible directions for future work.

## 2. Experimental Setup

### 2.1. Belt Grinding Mechanism

**Figure 1:**
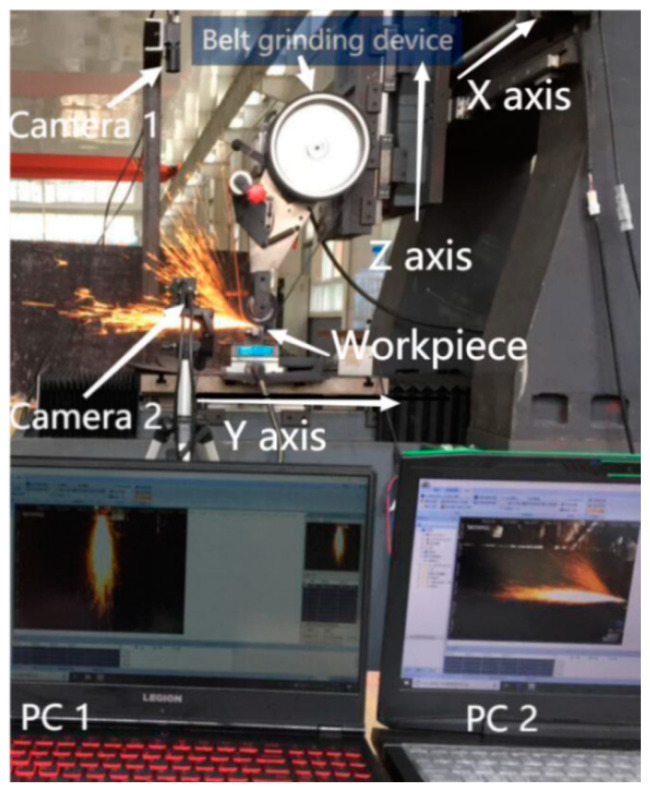
Belt grinding system.

An experimental platform was built to enable the study of spark images, as shown in Figure 1, consisting mainly of a three-axis machine tool, two high-speed CCD cameras and two computers. As shown in Figure 1, the workpiece selects GCr15 with a hardness of HRC58 and dimensions of 170 mm × 41 mm × 50 mm. The average roughness of GCr15 is 0.2 μm. The belt is tightly mounted on the *Z*-axis through the drive pulley, tensioning pulley, and contact pulley. With a motor speed of 0–5000 rpm, the belt is driven at a speed of 0–34 m/s. The contact wheel is rubber with a Shore A hardness of 85. The belt is made of corundum and has a width of 20 mm. Rotating at high speed cuts the workpiece better and generates a spark field.

A Beckhoff CX5130 embedded controller was selected as the experimental platform; the specific parameters are shown in Table 1. The controller was preinstalled with Windows operating system using the EtherCAT bus communication protocol.

The drive motor of the abrasive belt machine was a Y803-2 three-phase asynchronous motor with a rated speed of 2800 r/min. A Sunye brand CM800 vector frequency converter was selected to control the motor speed.

The spark image acquisition device used a MT-E200GC-T CMOS industrial camera; its specific parameters are shown in Table 2.

During the acquisition process, the CMOS camera was connected to the computer through the USB bus, and Mindvision software, which is capable of collecting images in real time, was used for image acquisition. In this experiment, the image acquisition frequency was set to 0.01 s, so 100 spark images were collected every 1 s. A hood was used during image acquisition in order to reduce the interference of other light in the experiment.

GCr15 with a hardness of HRC58 was used as the experimental specimen. A 60# brown corundum abrasive belt with a width of 20 mm was selected as the grinding abrasive belt in this study. A square workpiece of GCr15 material with a hardness of HRC58 and dimensions of 150 mm × 41 mm × 50 mm was used. The chemical composition is listed in Table 3.

In order to establish a spark-tracking model based on grinding, the method of controlling a single variable was used to change the material removal rate in the experiment. The belt speed range was 20 m/s–45 m/s, with increments of 0.25 m/s; a total of 100 sets of data were collected.

The experimental workpiece was fixed on the Y axis of the machine tool by two mounting holes. After cutting, the workpiece was fed in the Y direction according to the grinding parameters set in the experiment. Spark image collection started after the abrasive belt contacted the workpiece to generate sparks, ending after a grinding stroke ended. Each test piece was ground for 5 strokes, and the length of each stroke was 41 mm.

### 2.2. The Mechanism of Spark Generation

During the grinding process, due to the high-speed rotation of the abrasive belt, the abrasive grains on the belt cut the workpiece under pressure, and the cutting fragments on the workpiece are thrown out along the tangential direction of the contact wheel. As the debris carries a lot of heat, it generates sparks when it meets the air.

**Figure 2:**
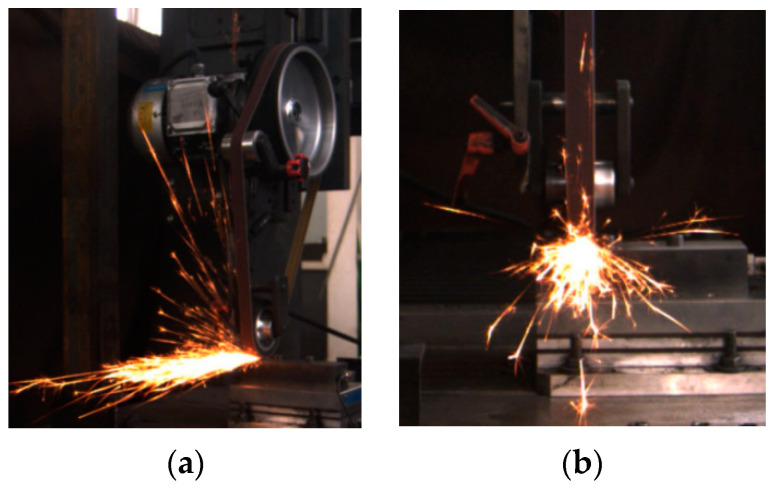
(**a**) Side spark image. (**b**) Frontal spark image.

In order to better process and study the relationship between spark images and the material removal rate, we set up two industrial CCD cameras directly above and to the side of the spark field, which were able to capture complete spark images at a frame rate of 100 Hz. PC1 saved and recorded the frontal spark images, and PC2 recorded and saved the side spark images. Figure 2a shows a side spark image acquired during the grinding process. Figure 2b shows a frontal spark image taken during the process. A total of 300 frontal spark images and 92 side spark images were collected during the complete grinding of a workpiece.

## 3. Image Preprocessing and Annotation

### 3.1. Image Preprocessing

**Figure 3:**
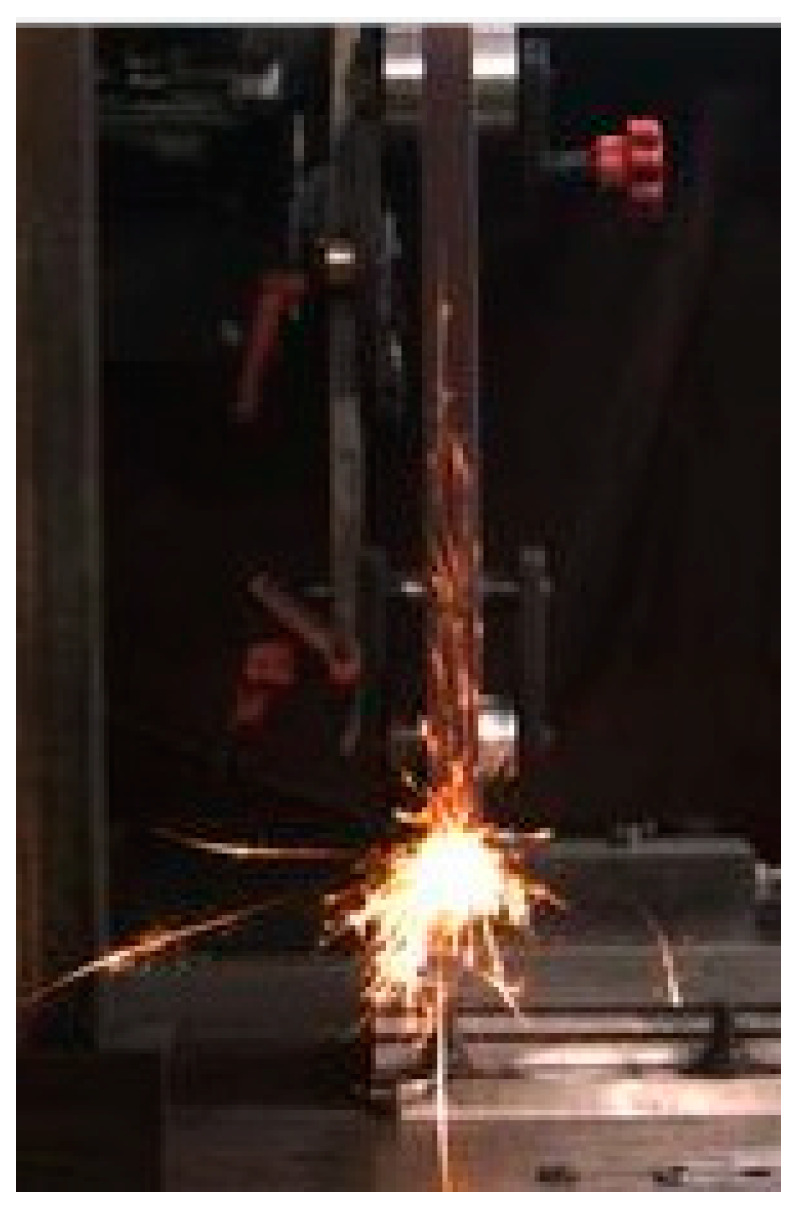
Cropped spark image.

The images captured by the CCD camera have dimension of 1600 × 1200 pixels, which far too many pixels, with a lot of useless background information. The images were preprocessed by cropping and scaling to retain useful spark information. The software processed all the images in the folder and converted them to 150 × 230 images, as shown in Figure 3. This preprocessing greatly increased the speed and efficiency of YOLO5 training.

### 3.2. Annotation of Images

**Figure 4:**
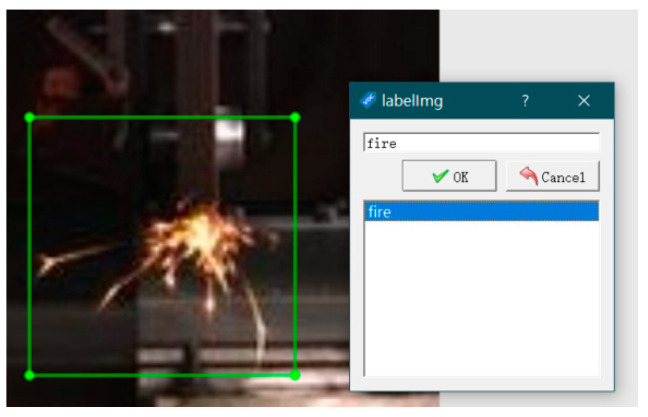
Image annotation.

Prior to training the images with YOLO5, the images should first be labelled with LabelImg. Because we were only detecting one target, we chose a “fire” label, as shown in Figure 4. After labelling all the images, YOLO5 can be used for training. We divided the training and test sets by placing the annotated frontal and side spark images in separate folders. The labelled image files were generated, along with the corresponding annotation files.

## 4. Methodologies

### 4.1. Overall Block Diagram

**Figure 5:**
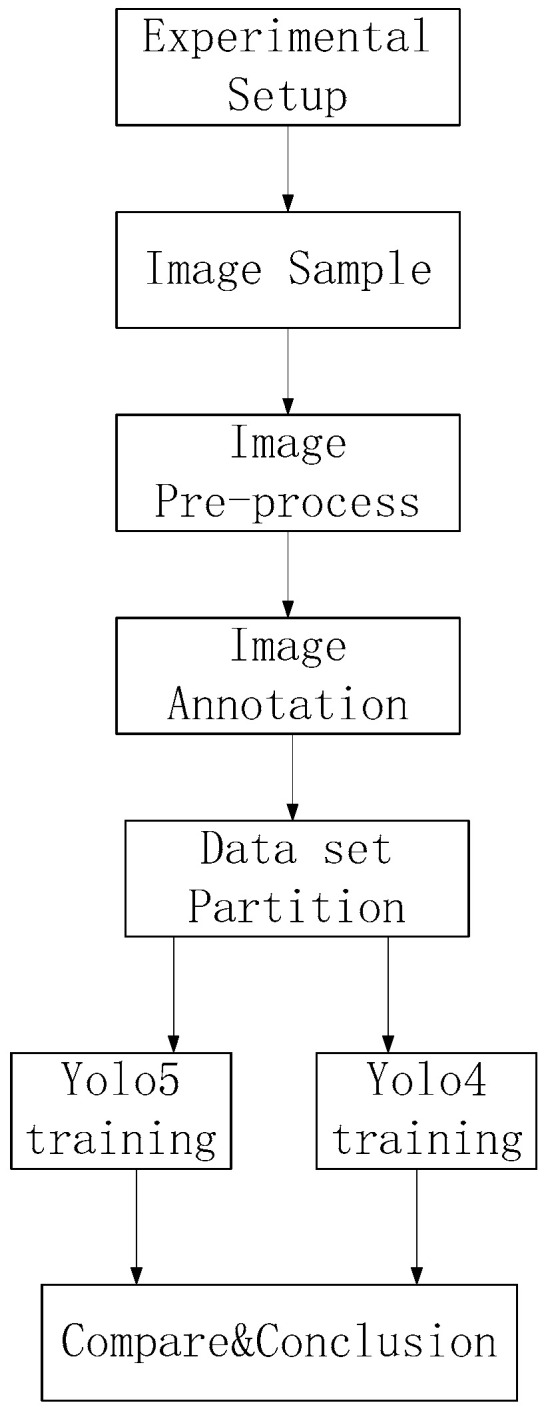
Overall flow chart of image target detection.

An overall flow chart of spark image processing is shown in Figure 5. First, an experimental platform was set up, as shown in Figure 1, and data acquisition was carried out for the frontal and side spark images. Then, the spark images were preprocessed. The target detection area was obtained by annotation with LabelImg. The spark images were divided into a training set and a test set, with 90% of images assigned to the training set and 10% assigned to the test set and trained with YOLO5 and YOLO4, respectively.

For the trials, a YOLOv5s-based deep learning network was used for detection of spark images, and 300 rounds of training were carried out on frontal spark images and side spark images to obtain the corresponding optimal models.

After completing the training with YOLOv5s, we retrained with YOLO4 to obtain the optimal models for each of the two images.

Lastly, the obtained models were validated separately using images from the test set to compare the network performance and verify the validity and real-time performance of the models.

### 4.2. YOLO5 Model

Since 2016, the You Only Look Once (YOLO) algorithm has passed through five generations; its most notable feature is its speed, which makes it particularly suitable for real-time target detection. YOLO5 is only 27 MB in size, while the YOLO4 model using the Darknet architecture is 244 MB in size, demonstrating that YOLO5 is nearly 90% smaller than YOLO4. Furthermore YOLO5 is the fastest version of this model, has a very lightweight model size, and is comparable to the YOLO4 benchmark in terms of accuracy.

**Figure 6:**
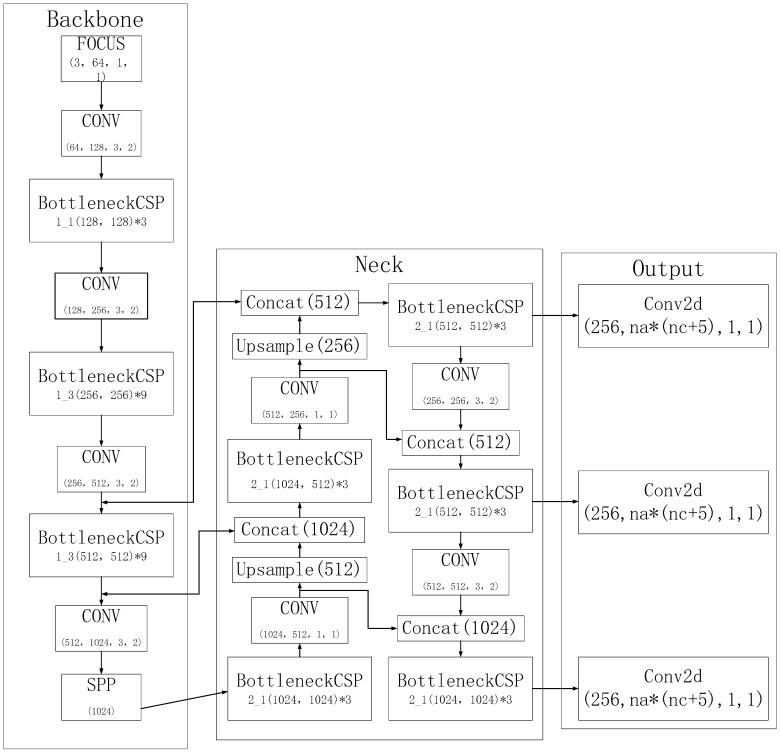
YOLO5 architecture.

The YOLO5 model consists of four versions—YOLOv5s, YOLOv5m, YOLOv5l, and YOLOv5x—with successively higher model parameters and performance. YOLO5 maintains the network structure of input, backbone, neck, and head outputs, as shown in Figure 6.

YOLOv5s is the smallest model in the YOLO5 family, with a width and depth of 0.33 and 0.5, respectively, while YOLOv5m, YOLOv5l, and YOLOv5x are based on the YOLOv5s model. The YOLO5 model maintains the mosaic data enhancement method of YOLO4, which randomly crops and stitches four images into one image as training data so that the input side can obtain information from four images at the same time, which enriches the image background information on the one hand and reduces the model’s reliance on batch size on the other hand. As an alternative, an adaptive anchor frame is proposed to calculate the optimal anchor frame value according to the difference relative to the training set.

YOLOv5s consists of four parts: input, backbone, neck, and output. On the input side, the image is automatically scaled, mosaic data enhancement is performed, and the best anchor frame value is automatically calculated. The other three parts are shown in Figure 6. The main modules shown in Figure 6 are described below.

#### 4.2.1. Focus Module

**Figure 7:**
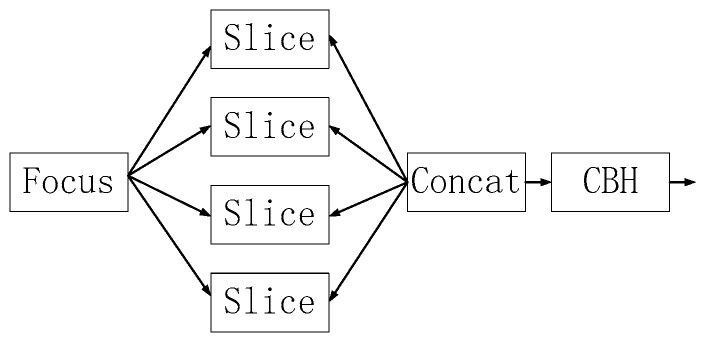
Structure of the focus module.

As shown in Figure 7, this module slices the image, expands the input channel by a factor of four, and convolves it once to obtain a downsampled feature map, reducing computational effort and increasing speed.

**Figure 8:**
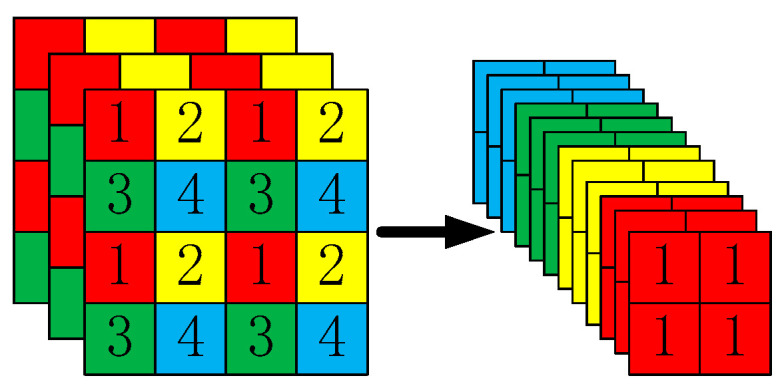
Focus slicing operation.

Taking YOLOv5s as an example, the original 640 × 640 × 3 image is fed into the focus structure, sliced into a 320 × 320 × 12 feature map, and convolved once to a 320 × 320 × 32 feature map. The slicing operation is illustrated in Figure 8.

As shown in Figure 8, the image is sliced before entering the backbone. The specific operation obtains a value for every other pixel in a picture, which was similar to adjacent downsampling, so that four pictures are obtained. The four images are complementary, and no information is lost. In this way, W and H information are concentrated in the channel space, and the input channel is expanded by 4 times. Compared with the original RGB 3-channel mode, the spliced pictures have 12 channels. Finally, the newly obtained image is subjected to a convolution operation, and a double downsampling feature map without information loss is obtained.

#### 4.2.2. BottlenetCSP Module

**Figure 9:**
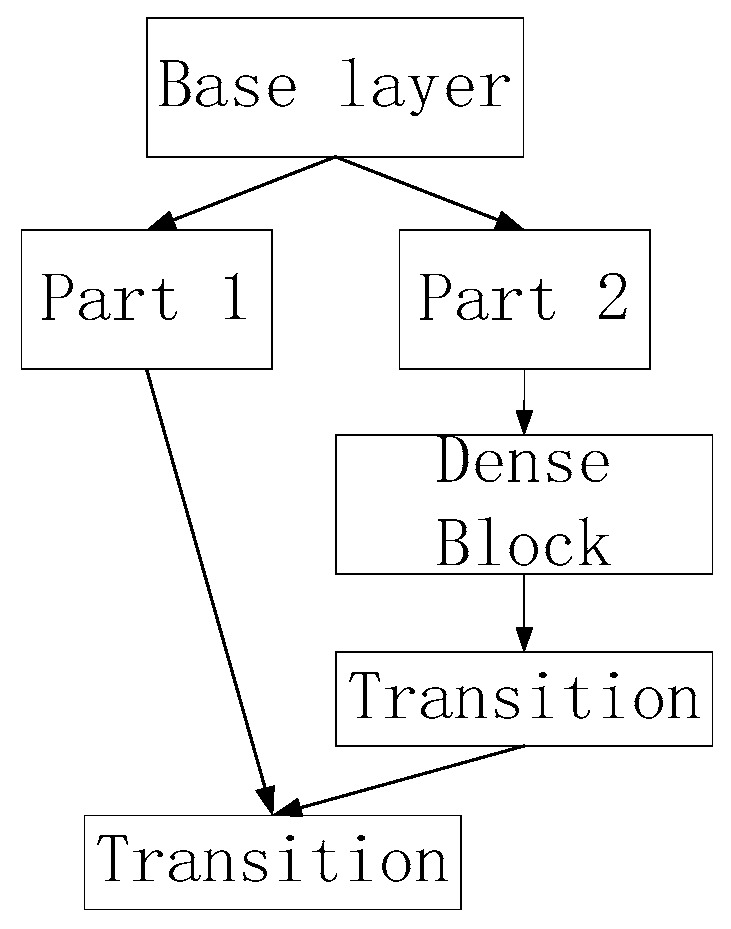
BottleneckCSP architecture.

The BottleneckCSP adopts the CSPDenseNet structure, as shown in Figure 9. According to the idea of cross-layer connectivity, partial cross-layer connections are made, and features from different layers are fused to obtain a richer feature map, both of which increase the depth of the network and save computational effort.

#### 4.2.3. SPP Module

**Figure 10:**
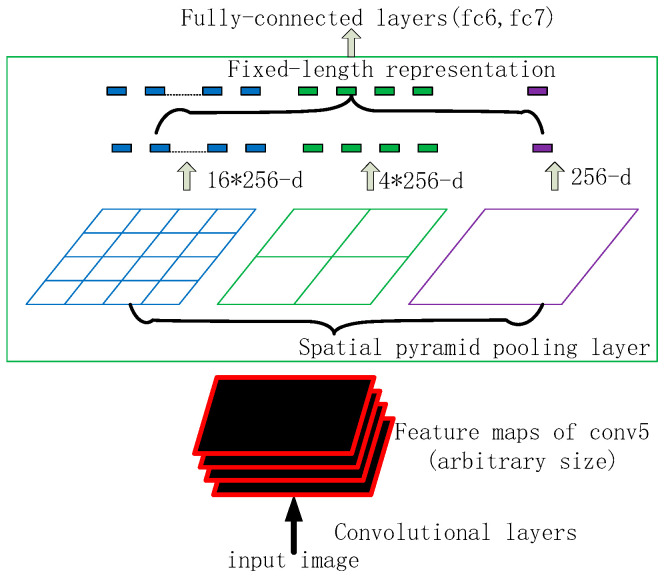
SPP structure.

The Spatial Pyramid Pooling Network (SPP-Net, a spatial pyramid pooling structure) was proposed by He et al. SPP-Net performs only one convolution operation on the image and uses the corresponding candidate box regions in the feature map by using pooling kernels of different sizes, as shown in Figure 10. Three pooling sizes are used, i.e., the feature map is divided into 44 and 22 with 11 sizes, and the feature map is taken in each grid using maximum pooling. With the introduction of the SPP module in YOLO5, the model can be trained on images of different sizes, enhancing the network’s generalisation capability.

SPP processing can effectively increase the receptive field and separate significant contextual features without losing the original detection speed.

#### 4.2.4. Output

Instead of YOLOv3’s IOU_Loss, the output layer uses GIOU_Loss as a loss function, adding a measure of intersection scale and alleviating the inability of IOU_Loss to optimise for cases in which two boxes do not intersect [22,23].

In comparison with IOU, GIOU solves the problem of non-differentiability of the loss function when the prediction frame does not intersect with the target frame (IOU = 0); on the other hand, when the two prediction frames are the same size and have the same IOU, the IOU loss function cannot distinguish between the intersection of the two prediction frames, which GIOU alleviates. The GIOU algorithm is as follows Algorithm 1 [24].
**Algorithm 1**: Generalised Intersection over Union [24]input: Two arbitrary convex shapes: A, B ⊆ S ∈ Rn output: GIoU 1 For A and B, find the smallest enclosing convex object C, where C ⊆ S ∈ R^n^ $IoU=\frac{\left|A\cap B\right|}{\left|A\cup B\right|}$          (1) $GIoU=IoU-\frac{\left|C\backslash \left(A\cap B\right)\right|}{\left|A\cup B\right|}$   (2)

## 5. Experiments and Discussion

### 5.1. Datasets

In this paper, we focus on the spark images generated during the grinding process, which can be divided into axonometric and frontal images. For target detection, we used rectangular boxes to mark the spark images, with only one object, i.e., “fire”.

During a complete workpiece polishing process, 300 frontal and 92 lateral spark images were collected. The resolution of the spark images captured by the HD industrial camera was 1600 × 1200. We divided the front and side spark image datasets into a training set and a test set, with 90% of the images were used for training and 10% used for testing [25,26].

### 5.2. YOLO5 Experimental Settings

(a) Training environment setup

The batch size was set to 32, with 300 epochs and an input image resolution of 640 × 640. Other parameter settings were consistent with the default settings of YOLOv5 [27]. The computer configuration used in the experiment is shown in Table 4.

(b) YOLO5 training parameter settings

The image resolution in the training set was 640 × 640. The number of “epochs” was set to 300, “batch size” was set to 16, momentum” was set to 0.98 to reduce the oscillation of the gradient descent, and the learning rate was set to 0.01. The other parameters were set to default values in YOLO5.

(c) YOLO5 Testing parameter settings

The image resolution in the testing set was 640 × 640. The IOU threshold for non-maximum suppression was set to 0.6. Other parameter settings were consistent with the default YOLO5 settings [28,29].

### 5.3. Training and Analysis of Results

**Figure 11:**
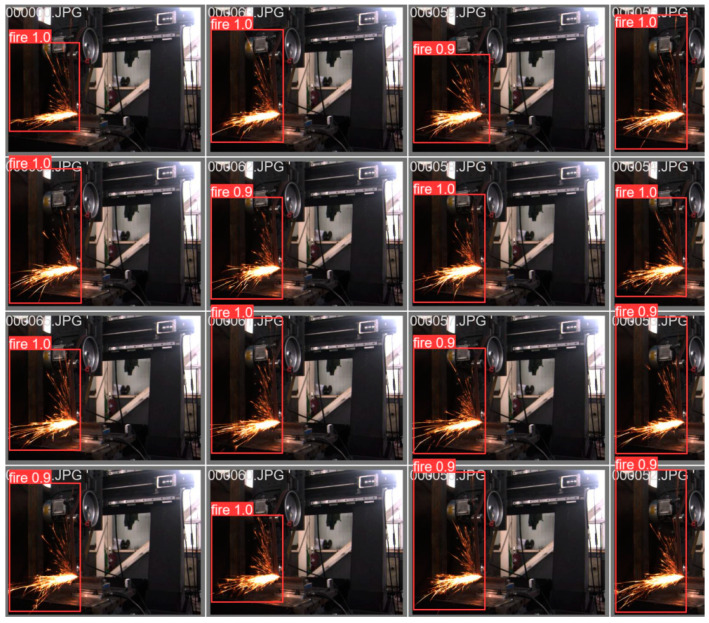
YOLO5 side spark image training results.

**Figure 12:**
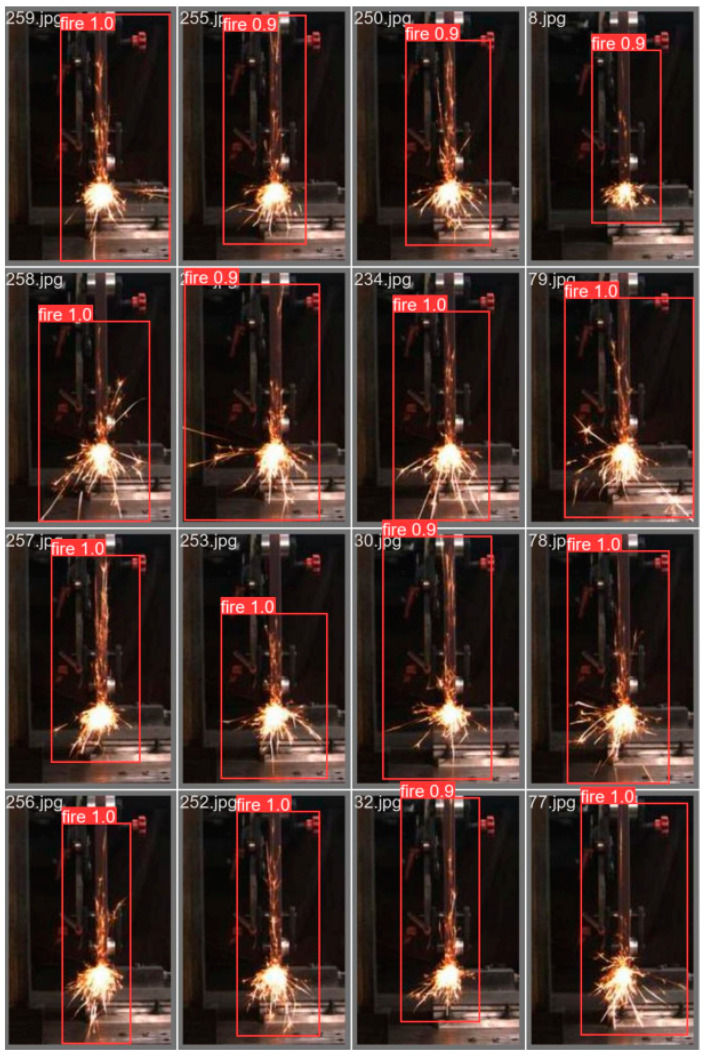
YOLO5 frontal spark image training results.

YOLO5 RTX3060 required 38 min to train 300 frontal spark images with 300 epochs and only 15 min to train 92 side spark images, also with 300 epochs, which is very fast. The final trained images are shown in Figure 11 and Figure 12.

**Figure 13:**
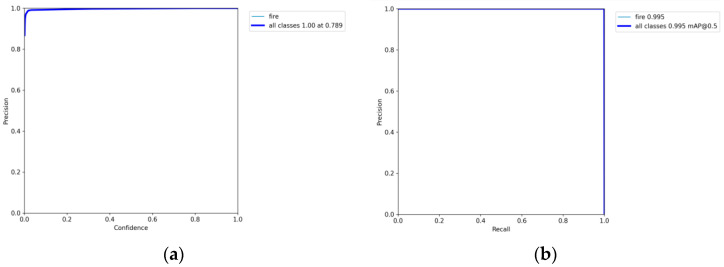
(**a**) Precision–confidence curve for YOLO5 frontal spark images. (**b**) Precision–recall curve of YOLO5 frontal spark images.

**Figure 14:**
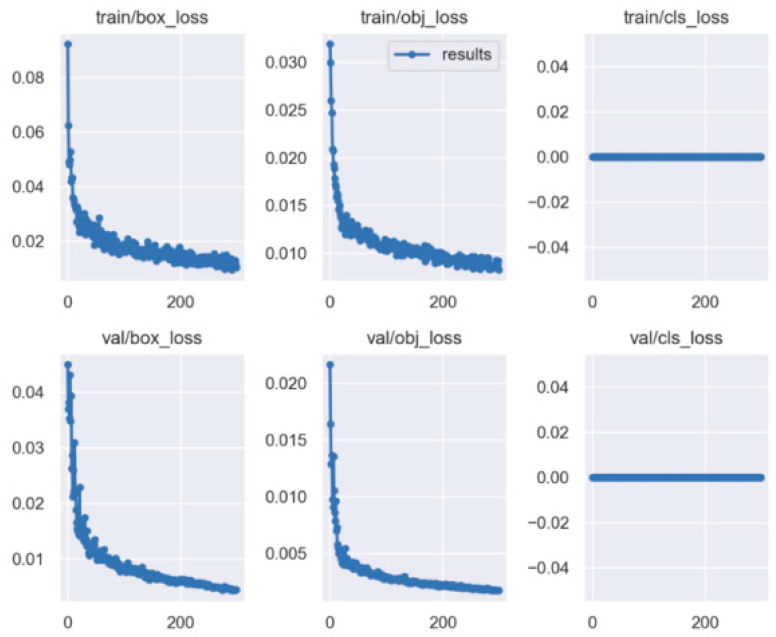
YOLO5 loss curve for positive spark image training.

After 300 rounds of training, the accuracy curve, PR curve, and LOSS curve of the frontal spark images were obtained, as shown in Figure 13 and Figure 14.

The loss is divided into three parts in the training process: cls_loss, box_loss, and obj_loss. cls_loss is used to supervise the category classification, box_loss is used to supervise the regression of the detection box, and obj_loss is used to supervise the presence or absence of objects in the grid [30,31].

**Figure 15:**
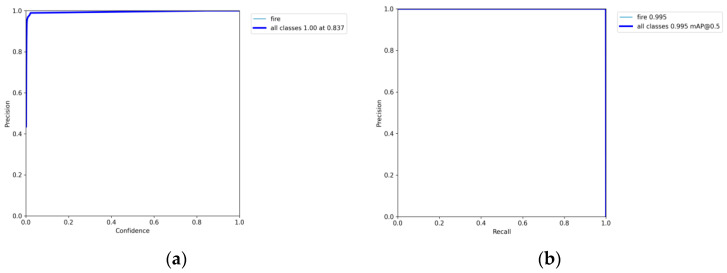
(**a**) Precision–confidence curve for YOLO5 frontal spark images. (**b**) Precision–recall curve for YOLO5 frontal spark images.

**Figure 16:**
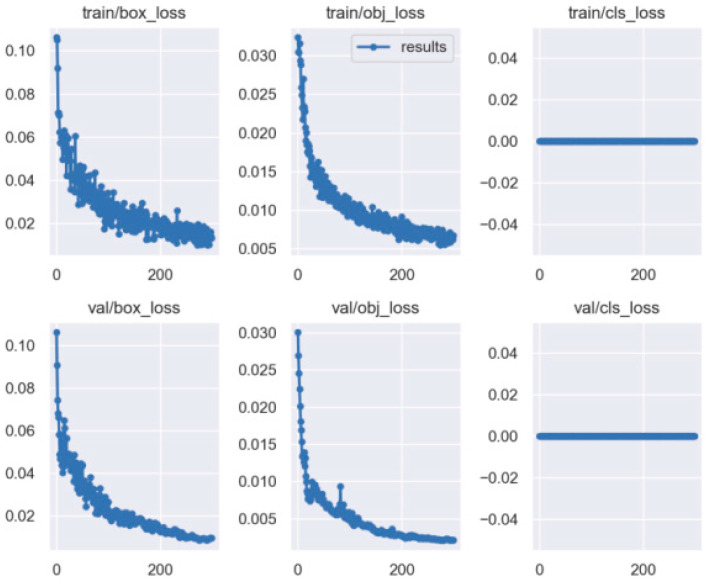
The loss curve of the YOLO5 for side spark images.

The same method was used to train the side spark images, and their corresponding accuracy, PR, and LOSS curves were also obtained, as shown in Figure 15 and Figure 16.

As shown by the above curves, the accuracy of the frontal spark images after training was up to 0.995 when mAP@0.5. After training, the side spark images, when mAP@0.5, the accuracy was up to 0.995. After 300 rounds of training, cls_loss, box_loss, and obj_loss all dropped below 0.01 according to the curves presented in Figure 16.

### 5.4. Forecasting and Analysis of Results

**Figure 17:**
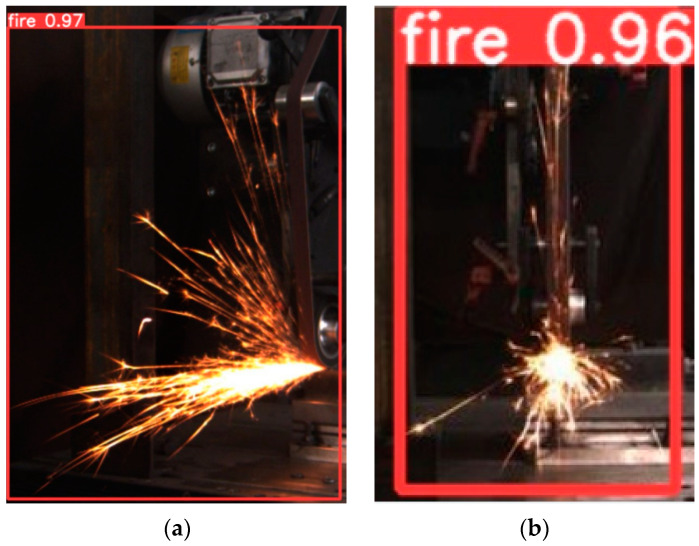
(**a**) Predicted results of YOLO5 side spark images. (**b**) Predicted results of YOLO5 frontal spark images.

Once the training was completed, the optimal model was obtained, which was used to predict the images of the test set, the results of which are shown in Figure 17.

As shown in Figure 17, when the optimal model generated by YOLO5 training was used to predict spark images, the accuracy of its target detection reached over 0.96, and the detection of a single image took 2 s.

### 5.5. YOLO4 Training and Prediction

For comparison of the advanced and fast YOLO5 detection algorithm, we also trained and predicted spark images with YOLO4. The corresponding computer software and hardware configurations are shown in Table 5.

The hardware configuration of the computers used for training and testing in YOLO4 is essentially the same as in YOLO5. The software configuration is slightly different; Cuda version 11.6 was used with the corresponding Cudnn version 8.4 and Darknet as the deep learning framework [32,33].

### 5.6. YOLO4 Training and Analysis of Results

**Figure 18:**
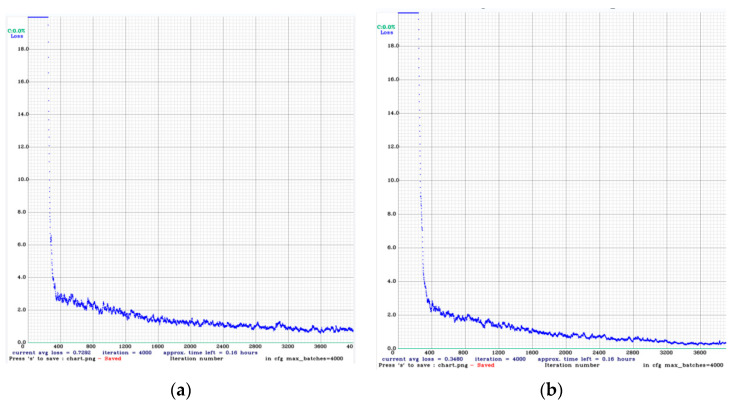
(**a**) YOLO4 Darknet training side spark image curve. (**b**) YOLO4 training frontal spark image profile.

It took close to 6 h for YOLO4 RTX3060 to train 300 frontal spark images with dimensions of 150 × 230 for a total of 4000 iterations [34]. Ninety-two side spark images were successfully trained by YOLO4 RTX3060 for a total of 4000 iterations, also taking close to 6 h. The final trained curves are shown in Figure 18.

**Figure 19:**
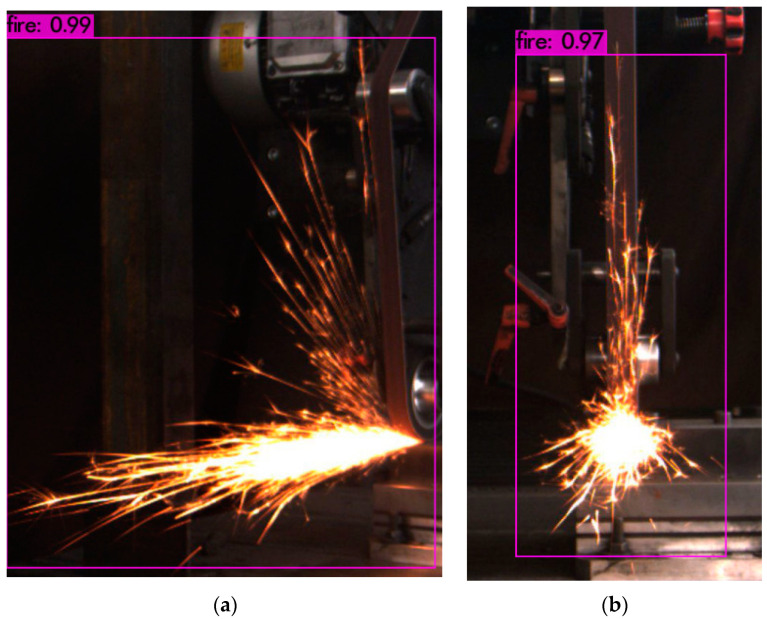
(**a**) YOLO4 side spark image prediction results. (**b**) YOLO4 frontal spark prediction.

In the curve presented in Figure 18, that the average loss is 0.348. After the training was completed, the optimised model was obtained, which we used to make predictions about the spark images, the results of which are shown in Figure 19.

A single spark image prediction took 5 s.

### 5.7. Discussion

After YOLO5 and YOLO4 training, we compared their performance metrics. The frontal and lateral sparks were treated separately for the training and test sets, and for performing metric comparison, we took the average value. The obtained results are shown in Table 6.

Compared to YOLO4, YOLO5 training is faster, with fewer rounds and higher accuracy. The optimised model generated after training is smaller in size and takes less time to predict, making it more suitable for real-time target detection than YOLO4. With more higher performance computer hardware configurations, online tracking and detection of spark images can be achieved.

## 6. Conclusions

In this paper, YOLO5 and YOLO4 were used to perform target detection on images of sparks generated during abrasive belt polishing of workpieces. The following conclusions can be drawn from the research process described above.

(1) YOLO5 was used for spark recognition in this study. The optimal model obtained after training is able to track the spark image area quickly and accurately, and with a more higher performancecomputer hardware configuration, even faster spark image recognition and detection can be achieved.
(2) Compared to YOLO4, the YOLO5 model has the advantage of high detection accuracy and high interference immunity. It can achieve good recognition under natural conditions, such as backlight or a dim machine tool processing environment, and can accurately identify and locate the spark image target.
(3) The small size of the YOLO5 model has better potential for portability than YOLO4, and with a more higher performance computer hardware configuration, the speed of target detection can reach the ms level, which is sufficient for real-time tracking of spark images. This work lays the foundation for future research on the automatic segmentation of spark images and the relationship between material removal rate and spark images.

Further research will be carried out in the future, mainly to investigate the following aspects:

(1) Segmentation of a complete spark image from the spark image area detected by YOLO5.
(2) Investigation of the relationship between the material removal rate and spark images;
(3) Establishment of a prediction model accounting for the relationship between the spark image and the material removal rate to realize automatic control of the grinding process.

## Figures and Tables

**Table 1:** Beckhoff CX5130 controller hardware parameters.

Content List	Performance
MCU	INTEL E3827
Memory	4 GB DDR3
Interface	2 × RJ45 10/100/1000 Mbit/s, 1 × DVI-I, 4 × USB2.0, 1×
System	Windows Embedded Compact 7
Power	20 w

**Table 2:** The main performance indicators of industrial cameras.

Performance	Parameter
Industrial camera model	HT-GE200GC
Sensor type	Colour COMS industrial camera
Pixels	2 million
Resolution	1200 × 1600
Lens sight distance	6–12 mm
Angle	28–53
Size	ϕ32 (mm) × 41 (mm)

**Table 3:** Chemical composition (wt%) and physical properties of the GCr15 workpiece.

Element	C	Cr	Mn	Si	P	S	Mo	Fe
Content	0.95–1.05	1.40–1.65	0.25–0.45	0.25–0.35	≤0.025	≤0.025	≤0.1	Other

**Table 4:** Computer configuration for YOLO5.

Project	Content
CPU	6core 11th Gen USA Intel(R) Core(TM) i5-11400F
RAM	32 GB
GPU	USA NVIDIA RTX3060 12GB
Operating system	USA Microsoft Windows 10
CUDA	USA NVIDIA Cuda11.3
Data Processing	USA Google Python3.8.8
Deep learning framework	USA Facebook Torch1.12

**Table 5:** Computer configuration for YOLO4.

Project	Content
CPU	6core 11th Gen Intel(R) Core(TM) i5-11400F
RAM	32 GB
GPU	RTX3060 12 GB
Operating system	Windows 10
CUDA	Cuda11.6 with Cudnn8.4
Data processing	Python3.8
Deep learning framework	Darknet

**Table 6:** Performance comparison between YOLO4 and YOLO5.

Performance Indicator	YOLO4	YOLO5
Epochs	4000	300
Training time	6 h	0.68 h
Testing time	5 s	2 s
mAP (%)	95.14	99.5
Recall (%)	88	82
FPS	4.2	7
Optimal model size (M)	227	14

## References

[1] Qi J.D., Chen B., Zhang D.H. (2020). Multi-information fusion-based belt condition monitoring in grinding process using the improved-Mahalanobis distance and convolutional neural networks. J. Manuf. Process..

[2] Pandiyan V., Shevchik S., Wasmer K., Castagnec S., Tjahjowidodod T. (2020). Modelling and monitoring of abrasive finishing processes using artificial intelligence techniques: A review. J. Manuf. Process..

[3] Pandiyan V., Caesarendra W., Tjahjowidodo T., Tan H.H. (2018). In-process tool condition monitoring in compliant abrasive belt grinding process using support vector machine and genetic algorithm. J. Manuf. Process..

[4] Gao K., Chen H., Zhang X., Ren X., Chen J., Chen X. (2019). A novel material removal prediction method based on acoustic sensing and ensemble XGBoost learning algorithm for robotic belt grinding of Inconel 718. Int. J. Adv. Manuf. Technol..

[5] Redmon J., Divvala S., Girshick R., Farhadi A. You only look once: Unifified, real-time object detection. Proceedings of the 2016 IEEE Conference on Computer Vision and Pattern Recognition (CVPR).

[6] Redmon J., Farhadi A. YOLO9000: Better, faster, stronger. Proceedings of the 2017 IEEE Conference on Computer Vision and Pattern Recognition (CVPR).

[7] Redmon J., Farhadi A. (2018). YOLOv3: An incremental improvement. arXiv.

[8] Bochkovskiy A., Wang C.-Y., Liao H.-Y.M. (2020). YOLOv4: Optimal Speed and Accuracy of Object Detection. arXiv.

[9] Liu W., Anguelov D., Erhan D., Szegedy C., Reed S., Fu C.Y., Berg A.C. (2016). SSD: Single shot multibox detector. Computer Vision-ECCV 2016.

[10] Girshick R., Donahue J., Malik T.D.J., Berkeley U. (2014). Rich feature hierarchies for accurate object detection and semantic segmentation Tech report (v5). arXiv.

[11] Girshick R. (2015). Fast R-CNN. arXiv.

[12] Ren S., He K., Girshick R., Sun J. (2016). Faster R-CNN: Towards Real-Time Object Detection with Region Proposal Networks. arXiv.

[13] He K., Gkioxari G., Dollar P., Girshick R. (2018). Mask R-CNN. arXiv.

[14] Fu L., Gu W.-b., Ai Y.-b., Li W., Wang D. (2021). Adaptive spatial pixel-level feature fusion network for multispectral pedestrian detection. Infrared Phys. Technol..

[15] Lian J., Yin Y., Li L., Wang Z., Zhou Y. (2021). Small Object Detection in Traffific Scenes Based on Attention Feature Fusion. Sensors.

[16] Wenkel S., Alhazmi K., Liiv T., Alrshoud S., Simon M. (2021). Confifidence Score: The Forgotten Dimension of Object Detection Performance Evaluation. Sensors.

[17] Wang J., Wang N., Li L., Ren Z. (2020). Real-time behavior detection and judgment of egg breeders based on YOLO v3. Neural Comput. Appl..

[18] Arunabha, Roy M., Bose R., Bhaduri J. (2022). A fast accurate fine-grain object detection model based on YOLO4deep neural network. Neural Comput. Appl..

[19] Ren L.J., Zhang G.P., Wang Y., Zhang Q., Huang Y.M. (2019). A new in-process material removal rate monitoring approach in abrasive belt grinding. Int. J. Adv. Manuf. Technol..

[20] Wang N., Zhang G., Pang W., Ren L., Wang Y. (2021). Novel monitoring method for material removal rate considering quantitative wear of abrasive belts based on LightGBM learning algorithm. Int. J. Adv. Manuf. Technol..

[21] Wang N., Zhang G., Pang W., Wang Y. (2021). Vision and sound fusion-based material removal rate monitoring for abrasive belt grinding using improved LightGBM algorithm. J. Manuf. Process..

[22] Huaibo S., Yanan W., Yunfei W., Shuaichao L., Mei J. (2022). Camellia Fruit Detection in Natural Scene Based on YOLO v5s. Trans. Chin. Soc. Agric. Mach..

[23] Wenliang W., Yanxiang L., Yifan Z., Peng H., Shihao L. (2021). MPANet-YOLOv5: Multi-Path Aggregation Network for Complex Sea Object Detection. J. Hunan Univ. Nat. Sci..

[24] Rezatofifighi H., Tsoi N., Gwak J., Sadeghian A., Reid I., Savarese S. (2019). Generalized Intersection over Union: A Metric and A Loss for Bounding Box Regression. arXiv.

[25] Tian Y., Zhao D., Wang T. (2022). An improved YOLO Nano model for dorsal hand vein detection system. Med. Biol. Eng. Comput..

[26] Tajar A.T., Ramazani A., Mansoorizadeh M. (2021). A lightweight Tiny-YOLOv3 vehicle detection approach. J. Real-Time Image Process..

[27] Zhang X., Chen H., Xu J., Song X., Wang J., Chen X. (2018). A novel sound-based belt condition monitoring method for robotic grinding using optimally pruned extreme learning machine. J. Mater. Process. Tech..

[28] Gai R., Chen N., Yuan H. (2021). A detection algorithm for cherry fruits based on the improved YOLO-v4 mode. Neural Comput. Appl..

[29] Ting Z.F. (2021). Research on Target Detection System of Basketball Robot Based on Improved YOLOv5 Algorithm.

[30] He K., Zhang X., Ren S., Sun J. (2015). Deep Residual Learning for Image Recognition. arXiv.

[31] He K., Zhang X., Ren S., Sun J. (2014). Spatial Pyramid Pooling in Deep Convolutional Networks for Visual Recognition. arXiv.

[32] Wang C.Y., Liao H.Y.M., Wu Y.H., Chen P.Y., Yeh I.H. (2020). CSPNet: A New Backbone that can Enhance Learning Capability of CNN. Proceedings of the IEEE/CVF Conference on Computer Vision and Pattern Recognition Workshops (CVPRW), Seattle, WA, USA, 14–19 June 2020.

[33] Liu S., Qi L., Qin H., Shi J., Jia J. Path aggregation network for instance segmentation. Proceedings of the IEEE Conference on Computer Vision and Pattern Recognition.

[34] Huang G., Liu Z., Laurens V., Weinberger K. (2018). Densely Connected convolutional Networks. arXiv.

